# A Bayesian Genomic Multi-output Regressor Stacking Model for Predicting Multi-trait Multi-environment Plant Breeding Data

**DOI:** 10.1534/g3.119.400336

**Published:** 2019-08-19

**Authors:** Osval A. Montesinos-López, Abelardo Montesinos-López, José Crossa, Jaime Cuevas, José C. Montesinos-López, Zitlalli Salas Gutiérrez, Morten Lillemo, Juliana Philomin, Ravi Singh

**Affiliations:** * Facultad de Telemática, Universidad de Colima, Colima, Colima, 28040, México,; † Departamento de Matemáticas, Centro Universitario de Ciencias Exactas e Ingenierías (CUCEI), Universidad de Guadalajara, Guadalajara, Jalisco, 44430, México,; ‡ International Maize and Wheat Improvement Center (CIMMYT), Apdo. Postal 6-641, Ciudad de México, 06600, México,; § Universidad de Quintana Roo, Chetumal, Quintana Roo, México,; ** Departamento de Estadística, Centro de Investigación en Matemáticas, Guanajuato, Guanajuato, 36023, México, and; †† Department of Plant Sciences, Norwegian University of Life Sciences, IHA/CIGENE, P.O. Box 5003, NO-1432 Ås, Norway

**Keywords:** Bayesian multi-output regressor stacking, multi-trait, multi-environment, GBLUP, genomic selection, breeding programs, regressor stacking, Genomic Prediction, GenPred, Shared Data Resources

## Abstract

In this paper we propose a Bayesian multi-output regressor stacking (BMORS) model that is a generalization of the multi-trait regressor stacking method. The proposed BMORS model consists of two stages: in the first stage, a univariate genomic best linear unbiased prediction (GBLUP including genotype × environment interaction GE) model is implemented for each of the L traits under study; then the predictions of all traits are included as covariates in the second stage, by implementing a Ridge regression model. The main objectives of this research were to study alternative models to the existing multi-trait multi-environment (BMTME) model with respect to (1) genomic-enabled prediction accuracy, and (2) potential advantages in terms of computing resources and implementation. We compared the predictions of the BMORS model to those of the univariate GBLUP model using 7 maize and wheat datasets. We found that the proposed BMORS produced similar predictions to the univariate GBLUP model and to the BMTME model in terms of prediction accuracy; however, the best predictions were obtained under the BMTME model. In terms of computing resources, we found that the BMORS is at least 9 times faster than the BMTME method. Based on our empirical findings, the proposed BMORS model is an alternative for predicting multi-trait and multi-environment data, which are very common in genomic-enabled prediction in plant and animal breeding programs.

Genomic selection (GS), first described by Meuwissen *et al.* (2001), is a plant and animal breeding methodology that is revolutionizing the selection of superior genotypes because it increases the rate of annual genetic gain by accelerating the breeding cycle and reducing the time and cost of phenotyping. GS assumes that a representative number of markers across the whole genome captures most of the diversity of the genome to estimate breeding values without the need to know where specific genes are located. In addition to markers, GS implementation relies on genomic-enabled prediction (GP) models that combine genomic (or pedigree) information and phenotypic data. Then the prediction equation of the implemented GP model is applied to a set of plants (or animals) for which genotypes (but not phenotypes) are available, to obtain the predicted breeding values (or phenotypes); finally, the best lines (plants or animals) are selected for breeding. For these reasons, it is obvious that GP models are a key element for successfully implementing GS. Considerable research has been done in recent years for improving the prediction accuracy of GP models; however, better prediction models to be able to implement the GS methodology more accurately are still lacking. Most of the research that has been done has been aimed at developing univariate-trait (UT) models and very little at developing multi-trait (MT) models. UT models are trained to predict the value of a single continuous (or categorical) phenotype in a testing dataset, while MT models are trained to predict at least two traits simultaneously.

In general, MT models are preferred over UT models because MT models: 1) represent complex relationships between traits more efficiently, 2) exploit not only the correlation between lines, but also the correlation between traits, 3) improve the selection index because they allow more precise estimates of random effects of lines and genetic correlation between traits, 4) can improve indirect selection because they increase the precision of genetic correlation parameter estimates between traits, and 5) improve the power of hypothesis testing better than UT models. For these reasons, MT models produce more accurate parameter estimates and better predictions than UT models, as documented by Montesinos-López *et al.* (2016, 2018a-b). For example, Schulthess *et al.* (2017) found empirical evidence that MT models improve parameter estimates. Calus and Veerkamp (2011), Jia and Jannink (2012), Jiang *et al.* (2015), Montesinos-López *et al.* (2016), He *et al.* (2016) and Schulthess *et al.* (2017) found that MT models outperform UT models in terms of prediction accuracy. However, these authors also found that when the correlation between traits is low using MT models, it is not advantageous (Montesinos-López *et al.*, 2016, 2018a-b), since the lower the degree of relatedness between traits, the lower the benefits of MT models (Montesinos-López *et al.*, 2016, 2018a-b). In a recent review of statistical models for genomic-enabled prediction including G×E and for different heritability values, the authors (Crossa *et al.*, 2017) did not report very high genomic-enabled prediction accuracies. However, it is recognized that from the computational resources perspective, fitting MT models is much more demanding than fitting UT models.

Multi-trait models are also known as multivariate analyses in statistical literature, and due to their clear advantages over UT models, they have been applied for solving a great diversity of problems in areas like environmental science, education, chemistry, telecommunications, psychology, medicine, communications, engineering and food science, among others. However, the use of MT models is not as popular as the use of UT models due to the following reasons: 1) there is less software available for performing MT analyses, 2) fitting MT models is computationally intensive and much more demanding than fitting UT models, 3) MT models are complex, as traits and variables have different response patterns in different environments and therefore create very complex genotype×environment interactions (G×E), 4) MT results are based on more assumptions than UT results and may be difficult to assess and achieve, and 5) MT models increase the problems of convergence when they are fitted with classic methods like maximum likelihood or restricted maximum likelihood, among others.

Recent literature on MT models has emphasized the use of multi-output regression (also known as multi-target, multi-variate, or multi-response regression) that aims to predict multiple continuous variables using a set of input variables. The output variables may also be binary (multi-label classification) or discrete (multi-dimensional classification) (Borchani *et al.*, 2015; 2016). Several real-world applications of multi-output regression have been studied in ecological models for assessing concentrations of species in natural populations, and in chemometric models for inferring concentrations of several substances from multivariate calibration using multivariate spectral data. Obviously, these applications give rise to many challenges such as missing data, the presence of noise that is typical due to the multivariate nature of the phenomenon, and the inevitable compound dependencies between multiple variables. Multi-output regression methods have the advantage of providing realistic methods for modeling multi-output datasets by considering the underlying relationships between the output variables, and thus give a better representation and interpretation of real-world problems (Borchani *et al.*, 2015). A further advantage of multi-output regression approaches is that they may produce simpler models that are computationally very efficient compared to other multi-trait models.

Borchani *et al.* (2015; 2016) present the multi-output regression approach as a problem transformation method where the multi-output problem is transformed into independent single-output problems, each of which is solved using a single-output regression algorithm and finally concatenating all the predictions. However, the problem with this method is that the relationships among output variables are ignored and thus predicted independently, and this can certainly affect the prediction accuracy. However, Spyromitros-Xioufis *et al.* (2012; 2016) recently extended the multi-output regression problem transformation method to target the dependency of the variables by introducing a novel multi-output regression approach called multi-output regressor stacking (MORS) (also called stacked single-target). The MORS method consists of two stages: in the first stage, a certain number of independent single-target models are fitted; however, those values are not used as final predictions but rather included in a second training stage where a second set of meta models is learned. In other words, the multi-output prediction problem is transformed into several single-target problems where any regression model could be used (*e.g.*, a separate ridge regression for each variable, a regression tree, support vector regression, etc.). Then, according to Spyromitros-Xioufis *et al.* (2012; 2016), the second stage includes using as predictor variables the predictions of the target variables obtained from the first-stage model. The second-stage model is expected to correct the predictions of the first-stage model using the information from the first-stage model.

Recently, Montesinos-López *et al.* (2019) presented an R package for analyzing breeding data with multiple traits and multiple environments that is an improved version of the original BMTME of Montesinos-López *et al.* (2016). This improved version of BMTME used the matrix variate normal distribution of Montesinos-López *et al.* (2018c) and the appropriate priors of Montesinos-López *et al.* (2018a) and Montesinos-López *et al.* (2018b). Interestingly the R package of Montesinos-López *et al.* (2019) evaluates the prediction performance of multi-trait multi-environment data in a user-friendly way. Also this R package introduced the Bayesian multi-output regressor stacking (BMORS) functions that are considerably efficient in terms of computational resources. For large datasets, the BME() and BMTME() functions of the BMTME R package are very intense in terms of computing time; however functions BMORS() and BMORS_Env() of BMORS (also included in the BMTME package) are less intensive in terms of computing time and produce similar genome-based prediction accuracies as the BMTME.

Given the previous considerations in terms of model prediction accuracy and the necessary computing resources required to fit different univariate and multivariate models, the main objectives of this research were: (1) to extend the theory of the multi-output regressor stacking model (MORS) to a Bayesian framework in the context of genomic selection; the resulting model is called Bayesian multi-output regressor stacking (BMORS), (2) to apply the BMORS model to seven extensive maize and wheat datasets from plant breeding programs, (3) to compare the prediction accuracy of the BMORS model with the accuracy of the most popular univariate trait (UT) model: the genomic best linear unbiased prediction (GBLUP) model and its multivariate version, called the Bayesian multi-trait multi-environment (BMTME) model, and (4) to compare the computing resources employed by BMORS *vs.* BMTME and the GBLUP. This comparison will determine the usefulness of the BMORS model compared with other analytical options with much heavier computing time.

## Materials and Methods

### Implemented models

#### Multiple-environment genotype × environment Genomic Best Linear Unbiased Predictor (GBLUP) model:

This model is the one proposed and described by Montesinos-López *et al.* (2018a). To implement this model, the genomic relationship matrix (GRM) was calculated as suggested by VanRaden (2008). This model is the conventional GBLUP that includes genotype × environment interaction; it was implemented in the Bayesian Generalized Linear Regression (BGLR) R-package of de los Campos and Pérez-Rodríguez (2014). Briefly, the model can be described as follows

$$y_{ij}=E_{i}+g_{j}+gE_{ij}+e_{ij}$$ (1)

where $y_{ij}$ is the response of the j th line in the i th environment (i=1,2,…,I, j=1,2,…,J). $E_{i}\;$is the fixed effect of the ith environment, $g_{j}\;$denotes the random genomic effect of the jth line, with $g={\left(g_{1},\ldots ,g_{J}\right)}^{T}∼N\left(0,\sigma_{1}^{2}\;G_{g}\right),$ $\sigma_{1}^{2}$ is the genomic variance and $G_{g}$ is the genomic relationship matrix (GRM) and is calculated (VanRaden 2008) as $G_{g}=\frac{WW^{T}}{p}$, where p represents the number of markers and W is the matrix of markers. The $G_{g}\;$ matrix is constructed using the observed similarity at the genomic level between lines. Further, $gE_{ij}$ is the random interaction term between the genomic effect of the jth line and the ith environment; let $\mathrm{gE}={\left(gE_{11},\ldots ,gE_{IJ}\right)}^{T}∼N\left(0,\sigma_{2}^{2}\;I_{I}⊗G\right)$, where $\sigma_{2}^{2}$ is the interaction variance, and $e_{ij}$ is a random residual associated with the jth line in the ith environment distributed as $N\left(0,\;\;\sigma^{2}\right),$ where $\sigma^{2}$ is the residual variance.

Since this model was implemented under a Bayesian framework, next we provide the priors used for the parameters. For the beta coefficients of the environments we used a normal distribution with mean 0 and very large variance $10^{10}$, that is, $N\left(0,10^{10}\right).$ For the genomic variance component, $\sigma_{1}^{2}$, we used a scaled inverse Chi-square with shape and scale parameters, $v_{1}=5$ and $S_{1}=Var\left(Y\right)\times R^{2}\times \left(v_{\beta }+2\right)$, respectively, where the proportion of total variance $\left(R^{2}\right)$ was set to 0.5, and Var(Y) is the phenotypic variance of the response variable of the training set. Also, for the error variance component, we used a scaled inverse Chi-square with shape and scale parameters, $v_{e}=5$ and $S_{e}=Var\left(Y\right)\times 0.25\times \left(v_{\beta }+2\right),$ respectively.

#### Bayesian multiple-trait multiple-environment (BMTME) model:

The BMTME model is a multivariate version of the model given in equation (1) defined as follows:

$$Y=\mathrm{X\beta}+Z_{1}b_{1}+Z_{2}b_{2}+E$$ (2)

where Y is of dimension n×L, with L the number of traits and n=J×I, where J denotes the number of lines (genotypes) and I the number of environments, X is of order n×I, β is of order I×L, since $\beta =\{\beta_{il}\}$ for i=1,..,I and l=1,..,L, $Z_{1}$ is of order n×J, $b_{1}$ is of order J×L and contains the genotype×trait interaction term since $b_{1}=\{gt_{jl}\}$ where $gt_{jl}$ is the effect of genotype × trait interaction term for j=1,..,J and for j=1,..,L.$\;Z_{2}$ is of order n×IJ, $b_{2}$ is of order IJ×L and contains the genotype×environment×trait interaction, since $b_{2}=\{gEt_{jil}\}$, where $gEt_{jil}$ is the effect of genotype×environment×trait interaction for j=1,..,J, for i=1,..,I and for j=1,..,L. Vector $b_{1}\;$is distributed under a matrix-variate normal distribution with $NM_{J\times L}\left(0,G_{g},\Sigma_{t}\right),$ $\Sigma_{t}$ is the unstructured genetic (co)variance matrix of traits of order L×L, $b_{2}∼NM_{JI\times L}\left(0,\;\Sigma_{E}\;⊗G_{g},\Sigma_{t}\right)$, where $\Sigma_{E}\;$is an unstructured (co)variance matrix of order I×I and E is the matrix of residuals of order n×L with $E∼NM_{n\times L}\left(0,I_{n},R_{e}\right)$, where $R_{e}$ is the unstructured residual (co)variance matrix of traits of order L×L, and $G_{g}$ is the genomic relationship matrix described above.

The BMTME model resulting from equation (2) was implemented by Montesinos-López *et al.* (2016). It is important to point out that model (2) takes into account the genotype×environment terms in the ($Z_{2}b_{2})$ term, and for comparison purposes, we also ran the model in equation (2) but without the ($Z_{2}b_{2})$ term to study the effect on prediction performance with and without the genotype×environment term. The information of the Gibbs sampler for implementing the BMTME model is found in Montesinos-López *et al.* (2018b), and the priors of this model are given in detail in Montesinos-López *et al.* (2018c).

#### Bayesian multi-output regressor stacking (BMORS):

The proposed BMORS is a Bayesian version of the multi-trait regressor stacking method proposed by Spyromitros-Xioufis *et al.* (2012; 2016). The training of BMORS consists of two stages. In the first stage, L single univariate models are implemented using the GBLUP model given in equation (1) of Montesinos-López *et al.* (2018a), but instead of using the resulting predictions directly as the final output, the BMORS includes an additional training stage where a second set of L meta-models are implemented for each of the L traits under study using a Ridge regression model. Each meta-model is implemented with the following model:

$$y_{ij}=\beta_{1}{\hat{Z}}_{1ij}+\beta_{2}{\hat{Z}}_{2ij}+\ldots +\beta_{L}{\hat{Z}}_{Lij}+e_{ij}$$ (3)

where the covariates ${\hat{Z}}_{1ij},\;{\hat{Z}}_{2ij},\;\ldots ,{\hat{Z}}_{Lij}$, represent the scaled predictions of each trait obtained with the GBLUP model in the first-stage analysis and $\beta_{1},\ldots ,\beta_{L}$, the corresponding beta coefficients. The scaling of each prediction was performed by subtracting its mean ($\mu_{lij}$) and dividing by its corresponding standard deviation ($\sigma_{lij}$), that is, ${\hat{Z}}_{lij}$=$({\hat{y}}_{lij}-\mu_{lij})\sigma_{lij}^{-1}$, for each l=1,…,L. Therefore, the BMORS model contains as predictor information the scaled predictions of its response variables yielded by the first-stage models. In other words, the BMORS model is based on the idea that a second-stage model is able to correct the predictions of a first-stage model using information about the predictions of other first-stage models (Spyromitros-Xioufis *et al.*, 2012; 2016).

This method can be implemented with the BMORS() function in the BMTME package in the R statistical software (R Core Team 2017), as shown in **Appendices A** and **B.** The R package BMTME is available at the following link: https://github.com/frahik/BMTME and it is described in Montesinos-López *et al.* (2019). The priors for implementing this second step were: a normal with mean zero and variance $\sigma_{\beta }^{2}$ for the beta coefficients while for the variance component $\sigma_{\beta }^{2}$ and the error variance ($\sigma^{2})$ we used the scaled inverse Chi-squares with shape and scale parameters, $v_{\beta }=v_{1}=5$ and $S_{\beta }=S_{1}=Var\left(Y\right)\times R^{2}\times \left(v_{\beta }+2\right)$ respectively, where the proportion of total variance $\left(R^{2}\right)$ was set to 0.5 for the beta coefficients, equal to 0.25 for the error variance component, and Var(Y) is the phenotypic variance of the response variable of the training set. Finally, it is important to point out that the underlying method of inference for all the Bayesian methods implemented was based on Markov Chain Monte Carlo.

### Experimental datasets

A total of seven real datasets were analyzed, one dataset comprising maize lines and six datasets comprising wheat lines. All seven datasets include several environments and traits and were previously used in several studies.

#### Maize dataset 1:

This dataset has 309 maize lines and was used by Crossa *et al.* (2013) and Montesinos-López *et al.* (2016). Traits grain yield (GY), anthesis-silking interval (ASI), and plant height (PH) were evaluated and measured in three environments (E1, E2, and E3). Phenotypes are best linear unbiased estimates (BLUEs) obtained after adjusting for the experimental field design. After filtering for missing values and minor allele frequency, the number of single nucleotide polymorphisms (SNP) was 158,281. Filtering was first done by removing markers that had more than 80% of the maize lines with missing values, and then markers with minor allele frequency lower than or equal to 0.05 were deleted.

#### Wheat dataset 2:

This wheat dataset is composed of 250 wheat lines that were extracted from a large set of 39 yield trials grown during the 2013-2014 crop season in Ciudad Obregon, Sonora, Mexico (Rutkoski *et al.*, 2016). The measured traits were plant height (PTHT) recorded in centimeters, and days to heading (DTHD) recorded as the number of days from germination until 50% of spikes emerged in each plot, in the first replicate of each trial. Each trait was measured on 250 lines and three environments. Phenotypes were also BLUEs adjusted by the experimental design. Genomic information was obtained by genotyping-by-sequencing (GBS) and we used a total of 12,083 markers that remained after quality control. Three environments were included: bed planting with 2 irrigation levels (Bed2IR), bed planting with 5 irrigations levels (Bed5IR), and drip irrigation (Drip). Filtering also was done by removing markers that had more than 80% of the wheat lines with missing values, and markers with minor allele frequency lower than or equal to 0.01 also were deleted.

#### Wheat Iranian dataset 3:

This dataset consists of 2374 wheat lines evaluated in a drought environment (D) and a heat environment (H) at the CIMMYT experiment station near Ciudad Obregon, Sonora, Mexico, during the 2010-2011 cycle; it was used in Crossa *et al.* (2016). Two traits were measured: days to heading (DTHD) and plant height (PTHT). Both traits were measured in the two environments (Env1 and Env2) on the same 2374 lines. Of a total of 40,000 markers after quality control, we used 39,758 markers. In this dataset, markers with more than 80% of missing values were removed and markers with minor allele frequency lower than or equal to 0.05 were deleted.

#### Elite wheat yield trial (EYT) datasets 4-7:

These four datasets were planted at the Norman E. Borlaug Research Station, Ciudad Obregon, Sonora, Mexico, and correspond to elite yield trials (EYT) planted in four different cropping seasons with 4 or 5 environments in each cropping season. The lines that were included each year in each of the environments are the same, but those in different years are different lines. EYT dataset 4 was planted in 2013-2014 and contains 767 lines, EYT dataset 5 was planted in 2014-2015 and contains 775 lines, EYT dataset 6 was planted in 2015-2016 and contains 964 lines, and EYT dataset 7 was planted in 2016-2017 and contains 980 lines. An alpha lattice design was used and the lines were sown in 39 trials, each comprising 28 lines and two checks, with three replications and six blocks. In each dataset, four traits were recorded for each line: days to heading (DTHD, number of days from germination to 50% spike emergence), days to maturity (DTMT, number of days from germination to 50% physiological maturity or the loss of green color in 50% of the spikes), plant height (in centimeters, measured from the ground to the top of the spike), and grain yield (GY).

In EYT datasets 4 and 7, the lines under study were evaluated in 4 environments, while in EYT datasets 5 and 6, the lines were evaluated in 5 environments. For EYT dataset 4, the environments were: bed planting with 5 irrigations (Bed5IR), early heat (EHT), flat planting and 5 irrigations (Flat5IR), and late heat (LHT). For EYT dataset 5, the environments were: bed planting with 2 irrigation levels (Bed2IR), bed planting with 5 irrigations levels (Bed5IR), early heat (EHT), flat planting with 5 irrigation levels (Flat5IR) and late heat (LHT). For EYT dataset 6, the environments were: bed planting with 2 irrigation levels (Bed2IR), bed planting with 5 irrigations levels (Bed5IR), flat planting with 5 irrigation levels (Flat5IR), flat planting with drip irrigation (FlatDrip), and late heat (LHT). Finally, for EYT dataset 7, the four environments were: bed planting with 5 irrigations (Bed5IR), early heat (EHT), flat planting with 5 irrigation levels (Flat5IR) and flat planting with drip irrigation (FlatDrip).

Genome-wide markers for the 4,368 lines in the four datasets were obtained using genotyping-by-sequencing (GBS) (Elshire *et al.*, 2011; Poland *et al.*, 2012) at Kansas State University using an Illumina HiSeq2500. After filtering, 2,038 markers were obtained. Imputation of missing marker data were done using LinkImpute (Money *et al.*, 2015) and implemented in TASSEL (Bradbury *et al.*, 2007), version 5. Markers that had more than 50% missing data, less than 5% minor allele frequency, and more than 10% heterozygosity were removed, and 3,485 lines were obtained (767 lines in dataset 1, 775 lines in dataset 2, 964 lines in dataset 3 and 980 lines in dataset 4).

#### Evaluation of prediction performance:

The prediction accuracies of the three models under study (BMORS, BMTME and UT) were evaluated with 10 random cross-validations (CV): the whole dataset was divided into a training (TRN) and a testing (TST) set; 80% (or 60%) of the whole dataset was assigned to TRN and the remaining 20% (or 40%) was assigned to TST. Since we used sampling with replacement, one observation (line) may appear in more than one partition. The CV implemented mimics a prediction problem faced by breeders in incomplete field trials, where some lines may be evaluated in some, but not all, target environments. Since N=J×I denotes the total number of records per each available trait, then to select lines in the TST dataset, we fixed the percentage of data to be used for TST [PTesting = 20% (or 40%)]. Then 0.20 (or 0.4) ×N (lines) were chosen at random, and subsequently for each of these lines, one environment was randomly picked from I environments. The cells selected through this algorithm were allocated to the TST dataset, while the cells (ij) that were not selected were assigned to the TRN dataset. Lines were sampled with replacement if J<0.20(or 0.4)×N, and without replacement otherwise. The prediction accuracy was evaluated with the average Pearson’s correlation (APC) and mean arctan absolute percentage error (MAAPE) of the testing sets of the 10 random partitions that were generated with the implemented CV. It is important to point out that the first 3 datasets were implemented with 80% and 20% for TRN and TST, respectively, while the last 4 datasets were implemented with 60% and 40% for TRN and TST, respectively. It is important to point out that performance via cross-validation was based on the mean sample from the posterior distribution of predicted values. To make the models comparable in their prediction accuracy as well as on their computing time, exactly the same random cross-validations were used for the three models.

The MAAPE is computed as the arctan of the absolute value of the difference between the observed value minus the predicted value divided by the observed value. Its advantage is that it is defined in radians and therefore scale-free and can include observations with missing values. Another advantage is that it approaches Pi over 2 for dividing by zero.

### Data availability

All seven datasets (Maize dataset 1, Wheat dataset 2, Wheat Iranian dataset 3, EYT datasets 4-7), including phenotypic and genotypic data, can be downloaded from the following link: hdl:11529/10548141 (http://hdl.handle.net/11529/10548141).

## Results

The results of this paper are presented in seven sections, each of which corresponds to a different dataset. Genomic-enabled prediction accuracy is presented in Table 1 for each model (BMORS, BMTME, and UT), trait, and dataset combination as average Pearson correlations (APC) and mean arctan absolute percentage error (MAAPE) with their corresponding standard deviations. Table 2 shows the time in minutes for fitting the three models for each trait and dataset.

**Table 1:** Average Pearson’s correlation (APC), mean arctan absolute percentage error (MAAPE) and their standard deviation (SD) for each trait (grain yield, GY, Plant height, PH, anthesis silking interval, ASI, days to heading, DTHD, plant height, PTHT, days to maturity, DTMT) and each maize and wheat dataset under study for three models, BMTME, BMORS and UT

Dataset	Model	Trait	APC	SD	MAAPE	SD
Maize dataset 1	BMORS	ASI	0.4263	0.0569	0.4024	0.0261
Maize dataset 1	BMORS	GY	0.3449	0.0391	0.1142	0.0055
Maize dataset 1	BMORS	PH	0.4683	0.0279	0.0395	0.0013
Maize dataset 1	BMTME	ASI	0.4338	0.0509	0.3944	0.0228
Maize dataset 1	BMTME	GY	0.3504	0.043	0.1112	0.0051
Maize dataset 1	BMTME	PH	0.4502	0.0366	0.0392	0.0013
Maize dataset 1	UT	ASI	0.4281	0.0525	0.3918	0.0226
Maize dataset 1	UT	GY	0.3522	0.0368	0.1095	0.0054
Maize dataset 1	UT	PH	0.4787	0.0281	0.0385	0.0013
Wheat dataset 2	BMORS	DTHD	0.8533	0.0378	0.5625	0.0253
Wheat dataset 2	BMORS	PTHT	0.4657	0.0434	0.6023	0.0234
Wheat dataset 2	BMTME	DTHD	0.8716	0.0287	0.4633	0.0234
Wheat dataset 2	BMTME	PTHT	0.4782	0.0419	0.6013	0.0203
Wheat dataset 2	UT	DTHD	0.8557	0.0368	0.5405	0.0252
Wheat dataset 2	UT	PTHT	0.4617	0.044	0.5935	0.0228
Wheat Iranian dataset 3	BMORS	DTHD	0.5862	0.0166	0.0392	4.00E-04
Wheat Iranian dataset 3	BMORS	DTMT	0.4288	0.0224	0.0605	8.00E-04
Wheat Iranian dataset 3	BMTME	DTHD	0.5918	0.0155	0.0389	5.00E-04
Wheat Iranian dataset 3	BMTME	DTMT	0.5182	0.0217	0.056	8.00E-04
Wheat Iranian dataset 3	UT	DTHD	0.5863	0.0173	0.0393	4.00E-04
Wheat Iranian dataset 3	UT	DTMT	0.4854	0.0208	0.0569	8.00E-04
EYT dataset 4	BMORS	DTHD	0.8149	0.0105	0.0356	0.001
EYT dataset 4	BMORS	DTMT	0.7768	0.0131	0.023	6.00E-04
EYT dataset 4	BMORS	GY	0.4444	0.0224	0.0736	0.0021
EYT dataset 4	BMORS	Height	0.5455	0.0207	0.0381	7.00E-04
EYT dataset 4	BMTME	DTHD	0.8363	0.0075	0.0303	6.00E-04
EYT dataset 4	BMTME	DTMT	0.7973	0.012	0.0212	5.00E-04
EYT dataset 4	BMTME	GY	0.4311	0.0234	0.0734	0.0021
EYT dataset 4	BMTME	Height	0.5728	0.0156	0.0374	8.00E-04
EYT dataset 4	UT	DTHD	0.8161	0.0101	0.0341	8.00E-04
EYT dataset 4	UT	DTMT	0.7726	0.013	0.0228	5.00E-04
EYT dataset 4	UT	GY	0.4551	0.0231	0.0713	0.0018
EYT dataset 4	UT	Height	0.5483	0.0182	0.0378	7.00E-04
EYT dataset 5	BMORS	DTHD	0.8519	0.0154	0.0241	8.00E-04
EYT dataset 5	BMORS	DTMT	0.7928	0.0165	0.016	6.00E-04
EYT dataset 5	BMORS	GY	0.5589	0.0269	0.0694	0.0019
EYT dataset 5	BMORS	Height	0.5563	0.0232	0.0349	0.001
EYT dataset 5	BMTME	DTHD	0.8596	0.0132	0.0217	6.00E-04
EYT dataset 5	BMTME	DTMT	0.8112	0.0153	0.0149	6.00E-04
EYT dataset 5	BMTME	GY	0.5486	0.0257	0.0698	0.0019
EYT dataset 5	BMTME	Height	0.563	0.0244	0.0348	0.0011
EYT dataset 5	UT	DTHD	0.8519	0.0152	0.0237	7.00E-04
EYT dataset 5	UT	DTMT	0.7902	0.0159	0.016	6.00E-04
EYT dataset 5	UT	GY	0.5621	0.0271	0.0684	0.002
EYT dataset 5	UT	Height	0.5591	0.0244	0.0346	0.001
EYT dataset 6	BMORS	DTHD	0.8416	0.0104	0.0203	6.00E-04
EYT dataset 6	BMORS	DTMT	0.7292	0.0128	0.0161	3.00E-04
EYT dataset 6	BMORS	GY	0.4855	0.0206	0.0779	0.0025
EYT dataset 6	BMORS	Height	0.5093	0.0198	0.0434	9.00E-04
EYT dataset 6	BMTME	DTHD	0.8278	0.0095	0.0204	7.00E-04
EYT dataset 6	BMTME	DTMT	0.7525	0.0116	0.0151	3.00E-04
EYT dataset 6	BMTME	GY	0.5043	0.0195	0.0755	0.0022
EYT dataset 6	BMTME	Height	0.5077	0.0232	0.0425	8.00E-04
EYT dataset 6	UT	DTHD	0.8425	0.0101	0.0201	6.00E-04
EYT dataset 6	UT	DTMT	0.731	0.0127	0.016	3.00E-04
EYT dataset 6	UT	GY	0.489	0.0183	0.0774	0.0023
EYT dataset 6	UT	Height	0.514	0.0187	0.0424	9.00E-04
EYT dataset 7	BMORS	DTHD	0.8406	0.0098	0.0314	6.00E-04
EYT dataset 7	BMORS	DTMT	0.8576	0.0082	0.0174	4.00E-04
EYT dataset 7	BMORS	GY	0.512	0.0226	0.0686	0.0016
EYT dataset 7	BMORS	Height	0.3994	0.0258	0.048	0.0013
EYT dataset 7	BMTME	DTHD	0.8549	0.0092	0.0291	8.00E-04
EYT dataset 7	BMTME	DTMT	0.8674	0.0083	0.0163	5.00E-04
EYT dataset 7	BMTME	GY	0.468	0.0274	0.0714	0.0014
EYT dataset 7	BMTME	Height	0.4306	0.0276	0.0468	0.0014
EYT dataset 7	UT	DTHD	0.8403	0.0097	0.0307	7.00E-04
EYT dataset 7	UT	DTMT	0.8582	0.0079	0.0172	5.00E-04
EYT dataset 7	UT	GY	0.5132	0.024	0.0684	0.0016
EYT dataset 7	UT	Height	0.4006	0.0263	0.0468	0.0014

**Table 2:** Time in minutes for fitting models BMTME, BMORS and UT for each dataset. BMTME/BMORS is the ratio of the time for implementing BMTME model *vs.* BMORS; BMTME/UT is the ratio of the time for implementing BMTME *vs.* UT model and BMORS/UT is the ratio of the time for implementing BMORS *vs.* UT model

Dataset	BMTME	BMORS	UT	BMTME/BMORS	BMTME/UT	BMORS/UT
Maize dataset 1	1240.722	94.761	17.131	13.09	72.42	5.53
Wheat dataset 2	436.005	47.315	7.724	9.21	56.45	6.13
Wheat Iranian dataset 3	8569.043	831.946	151.263	10.30	56.65	5.50
EYT dataset 4	3175.780	277.315	52.305	11.45	60.72	5.30
EYT dataset 5	4884.547	420.583	80.833	11.61	60.43	5.20
EYT dataset 6	8040.091	657.109	127.833	12.24	62.90	5.14
EYT dataset 7	5713.488	436.653	82.771	13.08	69.03	5.28

### Maize dataset 1

**Figure 1:**
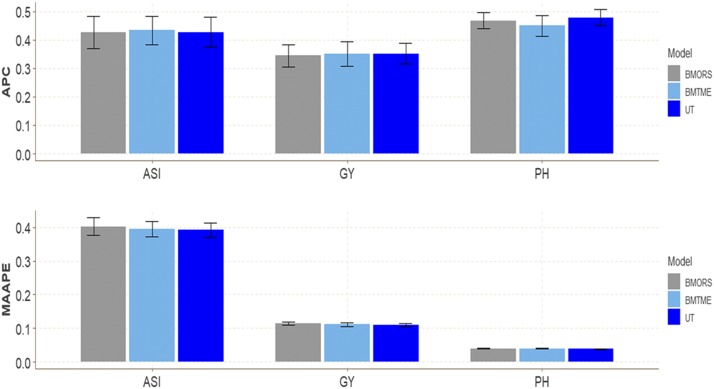
Maize dataset 1. Performance of three models (BMTME, BMORS and UT) under study in terms of average Pearson’s correlation (APC) and mean arctan absolute percentage error (MAAPE) for three traits: anthesis-silking interval (ASI), grain yield (GY) and plant height (PH) in three environments, E1, E2, and E3.

In this dataset we compare the prediction accuracy of the three models (BMORS, BMTME and UT). We did not find large differences in terms of prediction accuracy between the three models (Figure 1) across environments. The predictions in terms of APC for trait GY were 0.3449, 0.3504, and 0.3522 for models BMORS, BMTME, and UT. The APC for ASI was around 042-0.43 for the three models, and for trait PTHT, the range was between 0.4502 and 0.4787 (Table 1). In terms of MAAPE, the range of predictions was between 0.1095 and 0.1142 for trait GY. In summary, we found only slight differences in the APC and MAAPE of the three models for each trait.

For the whole dataset without cross-validation, we also compared the implementation time (computational resources) between the three methods and found that the slowest was the BMTME, while the fastest was the UT implemented in the BGLR package; BMTME was 13.09 and 72.42 times slower than UT and BMORS, respectively, while the BMORS method was only 5.53 times slower than UT (Table 2).

### Wheat dataset 2

**Figure 2:**
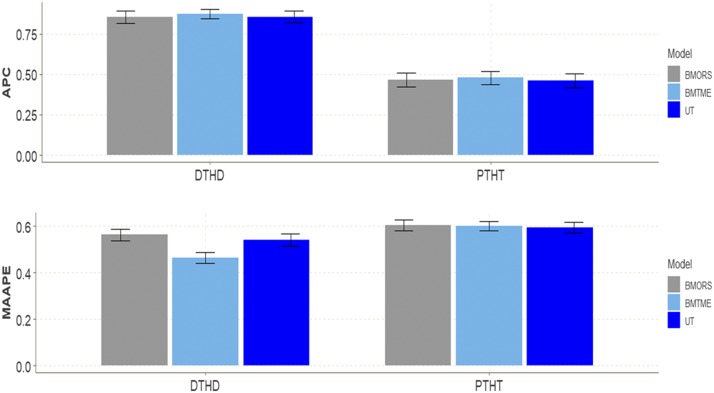
Wheat dataset 2. Performance of three models (BMTME, BMORS and UT) under study in terms of average Pearson’s correlation (APC) and mean arctan absolute percentage error (MAAPE) for two traits: days to heading (DTHD) and plant height (PTHT). Three environments were included: bed planting with two irrigation levels (Bed2IR), bed planting with 5 irrigations levels (Bed5IR), and drip irrigation (Drip).

In terms of APC, we did find some differences between the three models for both traits included in this dataset (Figure 2). The BMTME gave the highest APC for trait DTHD (0.8716) and PTHT (0.4782), followed by BMORS and UT with APC around 0.85 for DTHD. The range of predictions in terms of APC for trait DTHD was between 0.8533 and 0.8716, and between 0.4617 and 0.4782 for trait PTHT. However, for MAAPE, we found small differences in prediction accuracy only in trait DTHD, and the best model was the BMTME, which, on average, was better than the BMORS and UT models by 17.63% and 14.28%, respectively. The predictions for trait DTHD ranged between 0.4633 and 0.5625, while for trait PTHT, the predictions ranged between 0.5935 and 0.6023 (Table 1).

We also compared the implementation time (computational resources) between the three methods for the whole dataset, without cross-validation, and again we found that the slowest was the BMTME, while the fastest was BMORS. BMTME was 9.21 and 56.44 times slower than BMORS and UT, respectively, while the UT method was only 6.12 times slower than BMORS (Table2).

### Wheat Iranian dataset 3

**Figure 3:**
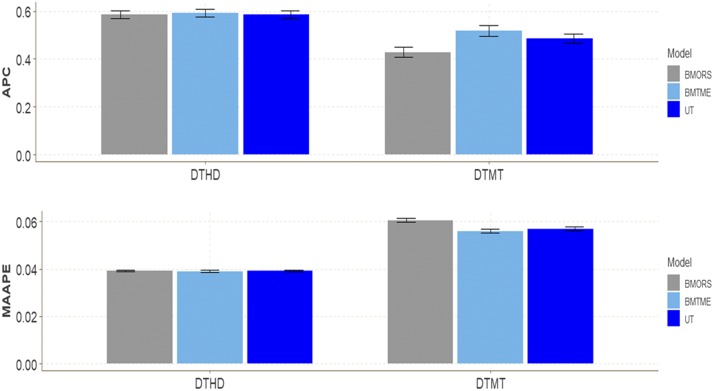
Wheat Iranian dataset 3. Performance of three models (BMTME, BMORS and UT) under study in terms of average Pearson’s correlation (APC) and mean arctan absolute percentage error (MAAPE) for two traits: days to heading (DTHD) and days to maturity (DTMT) in two environments (Env1 and Env2).

For this dataset we found more differences in APC between the traits for DTMT than for DTHD (Figure 3). In trait DTMT the best predictions were observed in model BMTME (0.5182) and the worst in model BMORS (0.4288) (Table 1). The average superiority of BMTME was 17.25% and 6.33% with regard to the BMORS and UT models, respectively. The predictions in terms of APC for trait DTHD ranged between 0.5862 and 0.5918, while for trait DTMT, they ranged between 0.4288 and 0.5182.

In terms of MAAPE, we only found differences between the three models for trait DTMT (Figure 3); the best predictions were observed in models BMTME and UT and the worst in model BMORS, but no significant differences were observed between the BMTME and UT models. The BMTME model was superior by 7.438% to the BMORS model. The predictions in terms of MAAPE for trait DTHD ranged between 0.039 and 0.039, while for trait DTMT, they ranged between 0.056 and 0.061 (Table 1).

Regarding the computer resources used when fitting each model to this dataset, the BMTME was the slowest in terms of implementation time, for it was 10.3 and 58.65 times slower than the BMORS method and UT model, respectively; the UT was 5.5 times faster than the BMORS (Table 2).

### EYT dataset 4

**Figure 4:**
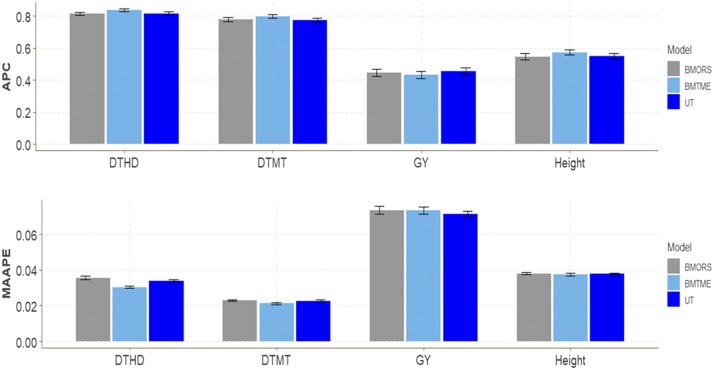
EYT dataset 4. Performance of three models (BMTME, BMORS and UT) under study in terms of average Pearson’s correlation (APC) and mean arctan absolute percentage error (MAAPE*)* for four traits: days to heading (DTHD), days to maturity (DTMT), grain yield (GY) and Height, evaluated in 4 environments: bed planting with 5 irrigations (Bed5IR), early heat (EHT), flat planting and 5 irrigations (Flat5IR), and late heat (LHT).

Figure 4 shows that although no large differences were found between the three models in DTHD, DTMT, and Height, the best predictions were observed in model BMTME with gains of 2.559%, 2.571% and 4.767% for traits DTHD, DTMT and Height, respectively (Table 1), compared to the BMORS model, and gains of 2.415%, 3.098% and 5.567%, respectively, compared to the UT model. However, for trait GY, the worst predictions were observed under the BMTME (0.4311), and models BMORS and UT were better than the BMTME model by around 3% and 5%, respectively. Values of APC ranged from 0.8149 to 0.8363 for DTHD, from 0.773 to 0.797 for DTMT, from 0.431 to 0.455 for GY, and from 0.546 and 0.573 for Height.

For the MAAPE criterion, there were differences between the three models in traits DTHD and DTMT, and the best model was the BMTME with gains of 14.888% (DTHD) and 7.826% (DTMT) over the BMORS model and gains of 11.144% (DTHD) and 7.018% (DTMT) for those traits over the UT model (Figure 4). The predictions under MAAPE ranged between 0.0303-0.0356 for DTHD, 0.0212-0.0230 for DTMT, 0.0713-0.0736 for GY, and 0.0374-0.0381 for Height (Table 1).

The implementation time between the three methods was as follows: the slowest was BMTME, the fastest was BMORS and UT was intermediate. BMTME was 11.45 and 60.72 times slower than BMORS and UT, respectively, while the UT method was only 5.30 times faster than BMORS (Table 2).

### EYT dataset 5

**Figure 5:**
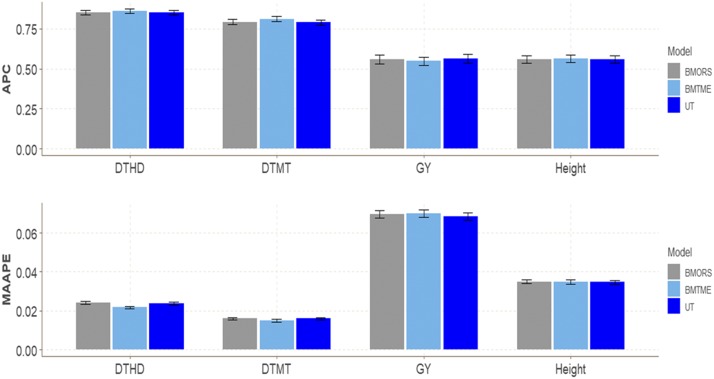
EYT dataset 5. Performance of three models (BMTME, BMORS and UT) under study in terms of average Pearson’s correlation (APC) and mean arctan absolute percentage error (MAAPE) for four traits (DTHD, DTMT, GY, Height) in five environments: bed planting with 2 irrigations (Bed2IR), bed planting with 5 irrigations (Bed5IR), early heat (EHT), flat planting with 5 irrigations (Flat5IR) and late heat (LHT).

No important differences between the three models in any of the four traits in terms of APC (Figure 5) were found. However, some differences were found for MAAPE in traits DTHD and DTMT with gains of 9.959% (DTHD) and 6.875% (DTMT) compared to the BMORS model, and gains of 8.439% (DTHD) and 6.875% (DTMT) compared to the UT model (Figure 5). The predictions in terms of APC for trait DTHD were around 0.85, between 0.7902 and 0.8112 for trait DTMT, between 0.5486 and 0.5621 for GY, and around 0.55-0.56 for Height (Table 1).

In terms of MAAPE, the range for trait DTHD was between 0.02170 and 0.02410, between 0.01490 and 0.01600 for trait DTMT, between 0.0684 and 0.0698 for trait GY and between 0.03460 and 0.03490 for trait Height. For this data set, the BMTME method was the slowest in implementation time, while the fastest was UT. BMTME was 11.61 and 60.43 times slower than BMORS and UT, respectively, while the BMORS method was only 5.2 times slower than UT (Table 2).

### EYT dataset 6

**Figure 6:**
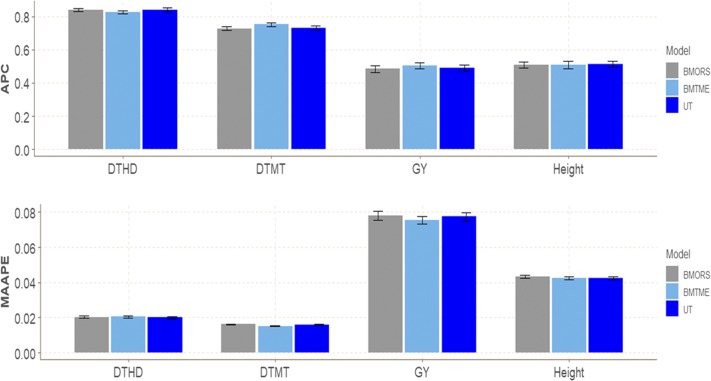
EYT dataset 6. Performance of three models (BMTME, BMORS and UT) under study in terms of average Pearson’s correlation (APC) and mean arctan absolute percentage error (MAAPE) for four traits (DTHD, DTMT, GY, Height) in five environments: bed planting with 2 irrigations (Bed2IR), bed planting with 5 irrigations (Bed5IR), flat planting with 5 irrigations (Flat5IR), flat planting with drip irrigation (FlatDrip), and late heat (LHT).

In terms of APC and MAAPE, we did not find large differences between the three models in any of the three traits (Figure 6). However, although large differences between models in terms of APC were not found for traits DMT and GY, the BMTME model was better than the BMORS and UT models for traits DTMT and GY, whereas for DTHD the best model was BMORS (0.8412) and for Height the best model was UT (0.514) (Table 1). In terms of MAAPE, the BMTME was superior by 6.211% (DTMT) and 3.081% (GY) compared to the BMORS, and by 5.625% (DTMT) and 2.455% (GY) compared to the UT model (Figure 6). For this dataset, the BMTME method was the slowest in implementation time, while the fastest was UT. BMTME was 12.24 and 62.89 times slower than BMORS and UT, respectively, while the BMORS method was only 5.14 times slower than UT (Table 2).

### EYT dataset 7

**Figure 7:**
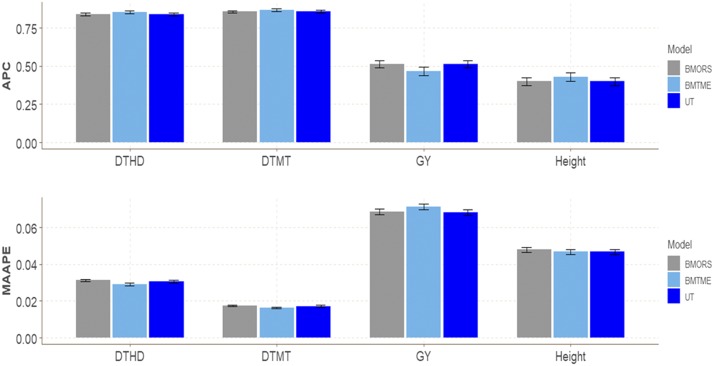
EYT dataset 7. Performance of three models (BMTME, BMORS and UT) under study in terms of average Pearson’s correlation (APC) and mean arctan absolute percentage error (MAAPE) for four traits (DTHD, DTMT, GY, Height) in 4 environments: bed planting system with 5 irrigations (Bed5IR), early heat (EHT), flat planting with 5 irrigations (Flat5IR) and flat irrigation with drip irrigation (FlatDr).

Figure 7 and Table 1 show that the best predictions were observed in model BMTME for traits DTHD (0.8549), DTMT (0.8674), and Height (0.4306). However, for trait GY, the worst predictions were observed under the BMTME model, and models BMORS and UT were better than the BMTME model by around 8%. In terms of MAAPE, we only found differences between the three models for traits DTHD and DTMT, and the best model was the BMTME compared to the BMORS and UT models. However, for trait GY, models BMORS and UT were the best, with a superiority of around 4% compared to the BMTME model, but no relevant difference was observed for this trait between the BMORS and UT models. Finally, concerning computational resources between the three methods for fitting the whole dataset, without cross-validation, we found that the slowest was the BMTME, while the fastest was UT. BMTME was 13.08 and 69.03 times slower than BMORS and UT, respectively, while the UT method was only 5.27 times slower than BMORS (Table 2).

## Discussion

In this study, we propose the BMORS model that basically consists of a two-stage univariate process, where a conventional univariate model is applied to the training set in the first stage, and those values are used in the second stage (for training and testing); here again a univariate model is implemented with the modified training set from which the final predictions are obtained. In the first stage, a GBLUP model was trained with features of the original dataset. Next, GBLUP’s first-stage predictions were used to build a meta-prediction model using the predictions of the first stage as features, but in the second stage, a Ridge regression method was used. This meta-prediction model was employed to obtain the final predictions.

When comparing the prediction ability of the BMORS model to that of UT and multivariate BMTME models, we found in most datasets, the BMTME model was better in terms of prediction accuracy than the UT and BMORS models. The predictions of the proposed BMORS were competitive since in general its prediction performance was similar to those of the BMTME and UT models, which may be due in part to the fact that the proposed BMORS model is a type of feature extraction technique that combines information from multiple predictive models to generate a new model. The main advantage of the proposed BMORS model is that it is considerably faster in terms of implementation time than the BMTME model. For example, in dataset 1, the BMORS method was 13.09 times faster than the BMTME, while in dataset 2, the BMORS method was 9.21 times faster than the BMTME, and in the remaining datasets (3 to 7), the BMORS was 10.3, 11.45, 11.61, 12.24 and 13.08 times faster than the BMTME, respectively, which allows its practical implementation. When comparing the implementation time, the superiority of the BMORS model is clear, since it was at least 9 times faster than the BMTME model. For this reason, although the prediction performance of BMORS is slightly lower than the prediction performance of the BMTME, this is compensated for by the fast implementation time even for moderate and large datasets, which is not possible with the BMTME model. In the proposed method, multiple weak learners can form one learner with (expected) higher prediction performance (strong learner), as opposed to a weak learner that usually performs slightly better than random guessing (Freund and Schapire 1997). However, the key difference between this technique and conventional ensemble techniques is that the multiple predictions are not from different models for the same trait or response variable, but for different correlated traits predicted with the same model. For this reason, the proposed model is called the featured extraction method, and not an ensemble technique. Also, in the second stage of the stacking process, we only included as predictors the predictions of the first stage, although some authors suggest including the original features plus the predictions of the first stage. It is important to point out that we also tested this option, but we got the worst predictions (results not presented). The proposed BMORS model can outperform the individual models (in the first stage) due to its smoothing nature and its ability to highlight each base model where it performs best and mistrust each base model where it performs poorly.

Our results are not in agreement with those of Li *et al.* (2017), who used the multi-trait regressor stacking method for time series prediction of drug efficacy and found that this method was considerably superior with regard to univariate analysis in most datasets they evaluated. The results of the present study are in agreement with those of Melki *et al.* (2017), who evaluated the multi-trait regressor stacking method under the support vector regression framework and found the trick of expanding the predictors in the second stage with the predictions of the target traits in the first stage in order to improve prediction accuracy. These authors also proposed making small modifications to this method in order to produce a performance similar to that of the original multi-trait regressor stacking method. Our results are also in agreement with those reported by Santana *et al.* (2017), namely, that using the outputs (predictions of the first stage) as additional prediction features in the second stage helps to increase prediction accuracy. However, it is important to point out that under the BMOR model, the increase in prediction accuracy was very modest only in some cases.

It is important to point out that the proposed BMORS method can be used for predicting material that has never been tested in the field, but of course we need to have the complete genotypic information of the lines to be predicted. Under this scenario, the implementation is the same as those described in this paper, but care should be taken when creating the design matrices to avoid problems in its implementation.

An advantage of the proposed BMORS model is that its implementation can be parallelized and implemented using current genomic-enabled prediction software. In this study, we implemented the BMORS model with the BGLR software developed by de los Campos and Pérez-Rodríguez (2014) using a two-stage process. It is important to recall that in the first stage, separate UT models were implemented for each trait with its corresponding training set, and predictions were made for each studied trait for both the training and testing sets, and then all predictions of the traits were used as covariates in a second-stage univariate analysis. With the same training set and with the predictions as covariates (predictions of the traits) of the first stage, the final predictions for each trait were performed. However, to successfully implement the BMORS model, the predictions of all traits used as covariates in the second stage should be standardized by subtracting their mean and dividing by their corresponding standard deviation.

Also it is important to point out that in the first stage, the proposed BMORS method allows parametric and non-parametric approaches, although here in the first stage we used the GBLUP with (co)variance structures of lines and lines × environments. However, other covariance structures are allowed that can be built with different types of kernels that can capture in a better way existing non-linearity in the data. Also, the first stage can be built using a pure parametric approach with existing software for genomic selection, like BGLR and others. However, in the second stage we used a Ridge regression approach, but other types of parametric and non-parametric approaches could be used; thus this method can be made more general in a very straightforward manner.

The implementation of the BMORS model is not restricted to a GBLUP in the first stage and Ridge regression in the second stage, which means that we can implement other types of models at each stage. For example, when the BMORS model is implemented in the BGLR statistical software, this allows using Bayesian Ridge regression, Lasso, Bayes A, Bayes B, RKHS, or a mixture of models at each stage. However, if the BMORS model is implemented in conventional restricted maximum likelihood (REML) software used for genomic-enabled prediction, then each stage can only be implemented under a REML framework. This shows that the application of the BMORS model is very flexible and can be implemented using the existing genomic-enabled prediction software. Breiman (1996) suggested that in the second stage, the beta (weights) coefficients of the predictor variables should be restricted to positive values; however, we tested this option by implementing the second stage with non-negative least squares using the library nnls (Lawson and Hanson 1995; Mullen and van Stokkum 2015), but the prediction accuracy was considerably worse. For this reason, both stages were implemented under a Bayesian framework without restriction on the beta coefficients.

One disadvantage of the proposed BMORS model compared to conventional multivariate analysis, is that it does not allow estimating genetic and residual covariances (and correlations) between traits. This is because the BMORS model is implemented in a two-stage process with separate UT models, which does not allow estimating these covariances. That is, the BMORS model is less interpretable than conventional multivariate analysis, which implies that the interpretation of beta coefficients makes no sense. However, for scientific studies where prediction is the main goal, not measuring the magnitude of the relationship between traits is not critical. It is important to point out that the BMORS method is appropriate for multi-trait analysis and should be preferred over the UT model when the correlation between traits is moderate or large; however, when this correlation is weak or close to zero, the UT model should be preferred because most of the time it will produce better predictions than the BMORS method and in less computing time.

Analyzing MT models in the context of GS is very challenging due to the size and complexity of the underlying datasets, which nowadays is common in many breeding programs; for these reasons, MT models require much more computational effort than UT models. However, due to the advantages of MT models when improving parameter estimates and prediction accuracy, and given the continued and dramatic growth of computational power, MT models play an increasingly important role in data analysis in plant and animal genomic-assisted breeding for selecting the best candidate genotypes early in time. For this reason, in plant and animal breeding the BMORS model is an attractive alternative for selecting candidate genotypes since it produces similar genomic-enabled predictions for multi-trait and multi-environment data as other statistical models but with much less computing time, since across the seven datasets we found that the BMORS model is at least 9 times faster than the BMTME method. Although the three methods evaluated in this article used the Markov Chain Monte Carlo method for Bayesian inference, the differences with regard to the implementation time that we found are mostly due to the model itself, and how the methods were implemented in each library.

## Conclusions

In this research we proposed a Bayesian multiple-output regressor stacking (BMORS) model applied in the context of genomic selection; it is the Bayesian version of the multi-output regressor stacking method proposed by Spyromitros-Xioufis *et al.* (2012; 2016). The BMORS model was implemented using a two-stage process, where in the first stage, a conventional univariate GBLUP model was implemented for its corresponding training set, while in the second stage a Ridge regression model was implemented for the same training set, but using as predictors the predictions in the first stage of the L traits under study. We found that the BMORS model was similar to the UT and BMTME models in terms of prediction accuracy, but in general, the BMTME model was the best. Finally, although more empirical evidence is needed to confirm the prediction performance of the BMORS model, the proposed BMORS model is another alternative for performing multi-trait genomic-enabled prediction in plant and animal breeding programs with significantly less computational time than is required for other genomic-enabled statistical packages.

## Appendix A

### R code for implementing the BMORS model in the BMTME package

rm(list=ls())

library(BMTME)

data(“WheatToy”) #########This data set is preloaded in the BMTME package#####

ls()

########Part a) Phenotypic data############################

phenoWheatToy <- phenoWheatToy[order(phenoWheatToy$Env, phenoWheatToy$Gid),]

rownames(phenoWheatToy)=1:nrow(phenoWheatToy)

head(phenoWheatToy)

#########Part b) Design matrix######################################

LG <- cholesky(genoWheatToy)

ZG <- model.matrix(∼0 + as.factor(phenoWheatToy$Gid))

Z.G <- ZG %*% LG

Z.E <- model.matrix(∼0 + as.factor(phenoWheatToy$Env))

ZEG <- model.matrix(∼0 + as.factor(phenoWheatToy$Gid):as.factor(phenoWheatToy$Env))

G2 <- kronecker(diag(length(unique(phenoWheatToy$Env))), data.matrix(genoWheatToy))

LG2 <- cholesky(G2)

Z.EG <- ZEG %*% LG2

Y <- as.matrix(phenoWheatToy[, -c(1, 2)])

head(Y)

############Part c) Cross validation and predictor########################

pheno <- data.frame(GID = phenoWheatToy[, 1], Env = phenoWheatToy[, 2],Response = phenoWheatToy[, 3])

ETA <- list(Env = list(X = Z.E, model = “BRR”), Gen = list(X = Z.G, model = ’BRR’), EnvGen = list(X = Z.EG, model = “BRR”))

CrossValidation <-CV.RandomPart(pheno, NPartitions = 10, PTesting = 0.2,set_seed = 123)

########### Part d) Implementing the BMORS model#############################

pm <-BMORS(Y, ETA = ETA, nIter = 15000, burnIn = 10000, thin = 2, progressBar = TRUE, testingSet = CrossValidation, digits = 4)

summary(pm)

boxplot(pm,select=”MAAPE”,las = 2)

## Appendix B

### Explanation of code of Appendix A and the output of the BMORS model

To be able to implement the BMORS method, it is necessary to install the BMTME library, which in addition to implementing the BMTME model also allows implementing the BMORS method. It is important to point out that the data used for illustrating the BMORS method is a toy wheat dataset called WheatToy that is preloaded in the BMTME package. For running the code given in Appendix A, you only need to copy and page this code in R and in this way you can run this in a straightforward way.

In Part **a) Phenotypic data**–we are ordering the data first by environment and then in each environment by the GIDs of lines. It is important to have the same order of the design matrices (environments, lines and genotype by environment) with those of the genomic relationship matrix.

In Part **b) Design matrix**–we create the design matrices of environments, lines and of genotypes by environments interaction. Here we also show how to incorporate the GRM into the design matrices of lines and genotype by environment interaction. At the end of this part the matrix of response variables (Y) is selected for which we will be implementing the BMORS method.

In part **c) Cross validation and predictor**—the R code is provided for creating the training-testing sets using the CV.RandomPart, for which an object called pheno is provided that contains information on environments, lines and response variables. In the CV.RandomPart() was specified 10 random partitions, with 20% for testing and 80% for training in each random partition. Then, also in this part of the code the predictor of the model is created as a list, which in this case contains three components corresponding to environments, lines and genotype by environment interactions. Finally, in part d) Implementing the BMORS model— the BMORS() function is used to implement the BMORS method that needs as input the information of the predictor (ETA), the matrix of response variables (Y), the number of iterations (nIter), the burn-in (burnIn), the thin value, and the information of the observations that will be used as testing (testingSet). The number of digits that should be used to print the results is also specified, and a TRUE or FALSE value function is specified in progressBar, to print or not to print the progress of the training process. And finally, with the summary(pm), we printed the output, which in this case is:

> summary(pm)

**Table:**

	Environment	Trait	Pearson	SE_Pearson	MAAPE	SE_MAAPE
1	Bed2IR	DTHD	0.8839	0.0606	0.3720	0.0398
2	Bed2IR	PTHT	0.6065	0.0867	0.7267	0.0444
3	Bed5IR	DTHD	0.9352	0.0197	0.4403	0.0590
4	Bed5IR	PTHT	0.2215	0.1534	0.3790	0.0543
5	Drip	DTHD	0.9341	0.0312	0.5445	0.0548
6	Drip	PTHT	0.6295	0.0733	0.7018	0.0278
